# Tandem E2F Binding Sites in the Promoter of the *p107* Cell Cycle Regulator Control p107 Expression and Its Cellular Functions

**DOI:** 10.1371/journal.pgen.1001003

**Published:** 2010-06-24

**Authors:** Deborah L. Burkhart, Stacey E. Wirt, Anne-Flore Zmoos, Michael S. Kareta, Julien Sage

**Affiliations:** 1 Departments of Pediatrics and Genetics, Stanford Medical School, Stanford, California, United States of America; 2 Cancer Biology Program, Stanford Medical School, Stanford, California, United States of America; 3 Institute for Stem Cell Biology and Regenerative Medicine, Stanford Medical School, Stanford, California, United States of America; Fred Hutchinson Cancer Research Center, United States of America

## Abstract

The retinoblastoma tumor suppressor (Rb) is a potent and ubiquitously expressed cell cycle regulator, but patients with a germline *Rb* mutation develop a very specific tumor spectrum. This surprising observation raises the possibility that mechanisms that compensate for loss of Rb function are present or activated in many cell types. In particular, p107, a protein related to Rb, has been shown to functionally overlap for loss of *Rb* in several cellular contexts. To investigate the mechanisms underlying this functional redundancy between Rb and p107 *in vivo*, we used gene targeting in embryonic stem cells to engineer point mutations in two consensus E2F binding sites in the endogenous *p107* promoter. Analysis of normal and mutant cells by gene expression and chromatin immunoprecipitation assays showed that members of the Rb and E2F families directly bound these two sites. Furthermore, we found that these two E2F sites controlled both the repression of *p107* in quiescent cells and also its activation in cycling cells, as well as in *Rb* mutant cells. Cell cycle assays further indicated that activation of *p107* transcription during S phase through the two E2F binding sites was critical for controlled cell cycle progression, uncovering a specific role for *p107* to slow proliferation in mammalian cells. Direct transcriptional repression of *p107* by Rb and E2F family members provides a molecular mechanism for a critical negative feedback loop during cell cycle progression and tumorigenesis. These experiments also suggest novel therapeutic strategies to increase the p107 levels in tumor cells.

## Author Summary

The retinoblastoma tumor suppressor Rb belongs to a family of cell cycle inhibitors along with the related proteins p107 and p130. Strong evidence indicates that the three family members have both specific and overlapping functions and expression patterns in mammalian cells, including in cancer cells. However, the molecular mechanisms underlying the functional differences and similarities among Rb, p107, and p130 are still poorly understood. One proposed mechanism of compensation is a negative feedback loop involving increased *p107* transcription in *Rb*-deficient cells. To dissect the mechanisms controlling *p107* expression in both wild-type and *Rb*-deficient cells, we have engineered inactivating point mutations into the E2F binding sites in the endogenous *p107* promoter using gene targeting in mouse embryonic stem cells. Gene expression and DNA binding assays revealed that these two sites are essential for the control of *p107* transcription in wild-type and *Rb* mutant cells, and cell cycle assays showed their importance for normal functions of p107. These experiments identify a key node in cell cycle regulatory networks.

## Introduction

The retinoblastoma gene *Rb* was initially identified as a prototypic tumor suppressor through its association with hereditary retinoblastoma; mutations in *Rb* or in genes that play a role in the regulation of Rb function are found in virtually all types of human cancers. The best-described function of Rb is to act as a transcriptional co-factor: Rb regulates the activities of numerous transcription factors and recruits chromatin remodeling complexes to control the expression of genes involved in the control of cell cycle progression, differentiation, and senescence. It is generally thought that the E2F family of transcription factors, consisting of both activating members (E2F1, E2F2, E2F3a) and some of the repressing members (E2F3b, E2F4, E2F5), are the most critical downstream mediators of Rb function in the control of cell cycle progression (reviewed in [1]–[3]).

Although Rb is expressed in nearly all cell types [4], patients and mice carrying heterozygous mutations for the *Rb* gene are not strongly predisposed to a broad range of tumors [5]–[9]. Perhaps most strikingly, conditional deletion of *Rb* in the mouse retina is insufficient to induce retinoblastoma [10]–[13], in sharp contrast to what is observed in human patients. After it was found that *Rb* is a member of a three-gene family, along with *p107* and *p130*, it was quickly hypothesized that one or both of these other Rb family members may be able to compensate for the absence of Rb in specific cell types. Indeed, *Rb/p107* and *Rb/p130* double knock-out mice develop retinoblastoma [10]–[14]. The ability of p107 to compensate for loss of Rb has since been observed in numerous cell types, beyond the mouse retina [13]–[19].

**Figure 1:**
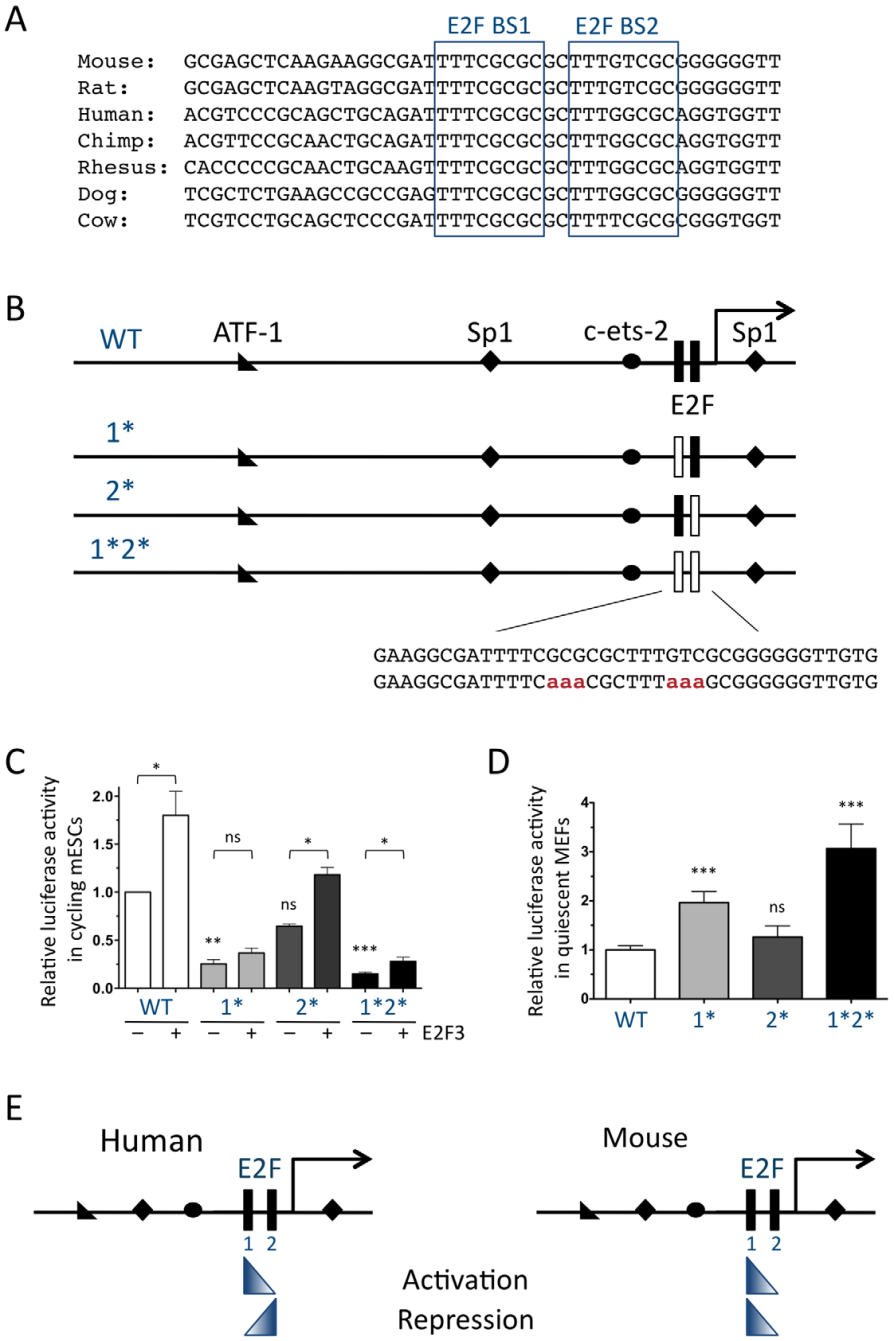
Regulation of the mouse *p107* promoter through E2F binding sites in reporter assays. (A) Conservation of the proximal *p107* promoter across mammalian species. The two tandem consensus E2F binding sites (BS1 and BS2) are each indicated by a box. (B) Schematic representation of wild-type (WT), p107-1*, p107-2*, and p107-1*2* luciferase vectors. Transcription factor binding sites contained in this promoter region, as identified by sequence analysis, are indicated, as is the transcription start site (arrow). Black rectangular boxes indicate E2F consensus sites; white boxes indicate E2F consensus sites that are mutated. The inset represents the mutations (aaa) introduced in each site. (C) Relative luciferase activity expressed by the four constructs, co-transfected with CMV-E2F3 (+) or empty pCDNA (−), in cycling mESCs. For statistical analysis, each mutant construct was compared to the wild-type one and the effect of E2F3 on each construct was analyzed. (n = 3) (D) Relative luciferase activity in quiescent MEFs. (n = 15) (E) Comparison of the models for the regulation of the human and mouse *p107* promoters by E2F based on reporter assays. Gradient triangles indicate the relative importance of each consensus E2F site to either activation or repression of *p107*.

The observation that the presence of p107 or p130 is able to suppress some phenotypes in the absence of Rb has raised the question of what molecular mechanisms underlie this compensatory activity. Of the three Rb family members, p107 is thought to be mostly regulated at the transcriptional level [20]–[22]; *p107* mRNA and protein levels are generally low in non-cycling cells, and expression increases as cells enter late G1 and S-phase [22], at a time when the protein is being functionally inactivated through phosphorylation. Because loss of Rb often results in increased levels of *p107* mRNA in mammalian cells [18], [23]–[26], an appealing model is that the absence of Rb directly affects *p107* transcription, resulting in genetic compensation rather than general functional redundancy. The 5′ regulatory region of the human *p107* gene contains two consensus E2F consensus binding sites (TTTSSCGC where S is G or C) [27] that are almost completely conserved among mammals (Figure 1A). These tandem E2F sites contribute to the appropriate cell-cycle induction of the human *p107* promoter in reporter assays [22]. In addition, E2F transcription factors directly bind to the *p107* promoter in a cell cycle-dependent manner suggesting a model in which activating E2Fs activate the *p107* promoter in late G1 and S while repressing E2Fs are associated with the *p107* promoter in G0 and early G1 [28], [29]. However, many of these reporter assays were performed using a minimal *p107* promoter transiently expressed in tumor cells lines, and may not fully recapitulate the regulation of the endogenous allele. In addition, chromatin immunoprecipitation (ChIP) experiments did not identify the exact sequences bound by E2F in the *p107* promoter, did not rule out that E2F could bind to other sequences, and did not determine if the two consensus sites were bound differently by different E2F family members. Moreover, from these experiments, it is still unclear how the cell cycle-dependent regulation of *p107* contributes to the cellular functions of p107. Finally, as Rb controls the activity of multiple transcription factors, whether the E2F binding sites or other transcription factor binding sites [30]–[32] (Figure 1B) mediate the repressive effects of Rb on the *p107* promoter is still unknown.

Traditional knockout studies, which delete an entire gene and modify multiple functional interactions in cells, may often obscure the importance of individual regulatory loops. To investigate the mechanisms underlying transcriptional regulation of *p107 in vivo*, we used gene targeting in mouse embryonic stem cells (mESCs) to engineer point mutations in the two E2F binding sites in the endogenous *p107* promoter. Disruption of this specific cis-acting node in the Rb/E2F network has allowed us to show that the tandem E2F binding sites in the *p107* promoter dynamically regulate *p107* levels in wild-type and Rb-deficient cells and control p107 function during S phase.

## Results

### Two consensus E2F binding sites in the mouse *p107* promoter contribute to activation and repression of transcription in reporter assays

To understand the functions of the tandem E2F consensus binding sites in the mouse *p107* promoter, we first generated a series of four luciferase constructs in which inactivating point mutations [33] were introduced into the two consensus E2F sites, either individually or together, into a construct containing 900 bp of the mouse *p107* promoter (Figure 1B). These constructs were transfected into wild-type mouse embryonic stem cells (mESCs), which contain high levels of activating E2F transcription factors as well as limited Rb family function, due to hyperphosphorylation of the Rb family proteins through high activity of Cyclin/Cdk complexes [34]–[36]. Because mESCs are rapidly cycling, we reasoned they would provide a good system to investigate the role of these consensus E2F sites in the activation of the *p107* promoter. In mESCs, the p107-1* construct, which contains a mutation in the more distal consensus E2F binding site, expressed less than half of the luciferase activity of the wild-type construct, whereas the p107-2* construct, which retains the more distal site but contains a mutant proximal site, expressed nearly 70% of the wild-type activity (Figure 1C). Simultaneous mutation of the two sites in the p107-1*2* construct resulted in even lower expression, suggesting that both sites contributed to some extent to the activation of the *p107* promoter, although the distal site appeared to mediate the majority of the activation. We also found that exogenous E2F3 efficiently enhanced the activity of the wild-type and the p107-2* constructs and to a much lesser extent that of the p107-1* and p107-1*2* mutant promoters (Figure 1C). These observations further suggested that the distal E2F consensus binding site mediated the majority of the activation of *p107* by E2F but did not exclude that some activation of *p107* could be mediated through the proximal site in this context.

We next investigated the potential role of E2F-mediated transcriptional repression in the control of *p107* transcription in quiescent cells, where *p107* levels are normally low. Because mESCs do not stably arrest in G0, the four reporter constructs were transfected into wild-type MEFs that were then serum-starved for 24 hours in order to induce cell cycle exit in G0. In this system, the p107-1* construct showed a significant trend towards increased reporter expression, suggesting that the distal site mediated a significant amount of repression of the *p107* promoter that could not be compensated by the presence of the proximal site (Figure 1D). However, the significant increase in luciferase activity found with the p107-1*2* construct over the p107-1* (Figure 1D) construct also suggested that both sites contributed to repression of *p107* to some extent in this context.

Together, these results are indicative of a model for the mouse *p107* promoter in which the distal site is most significant for both the activation and repression of the *p107* promoter, while the proximal site contributes to a lesser extent to both functions (Figure 1E, right). In contrast, previous experiments using the human promoter had generated a model in which the distal consensus E2F binding site had a more significant role in repression of *p107* while the proximal site was more important for activation [22], [27] (Figure 1E, left). This difference between the mouse and the human promoters could potentially result from a single polymorphism between the mouse and human sequences in the proximal consensus E2F binding site (Figure 1A) (see Discussion). Nevertheless, these results with plasmid reporters also underscore the fact that the two tandem E2F consensus sites may perform different functions in different contexts, raising the question of their respective roles in the control of the endogenous *p107* gene.

### E2F transcription factors control endogenous *p107* expression in mESCs via tandem consensus E2F binding sites in the proximal promoter region

**Figure 2:**
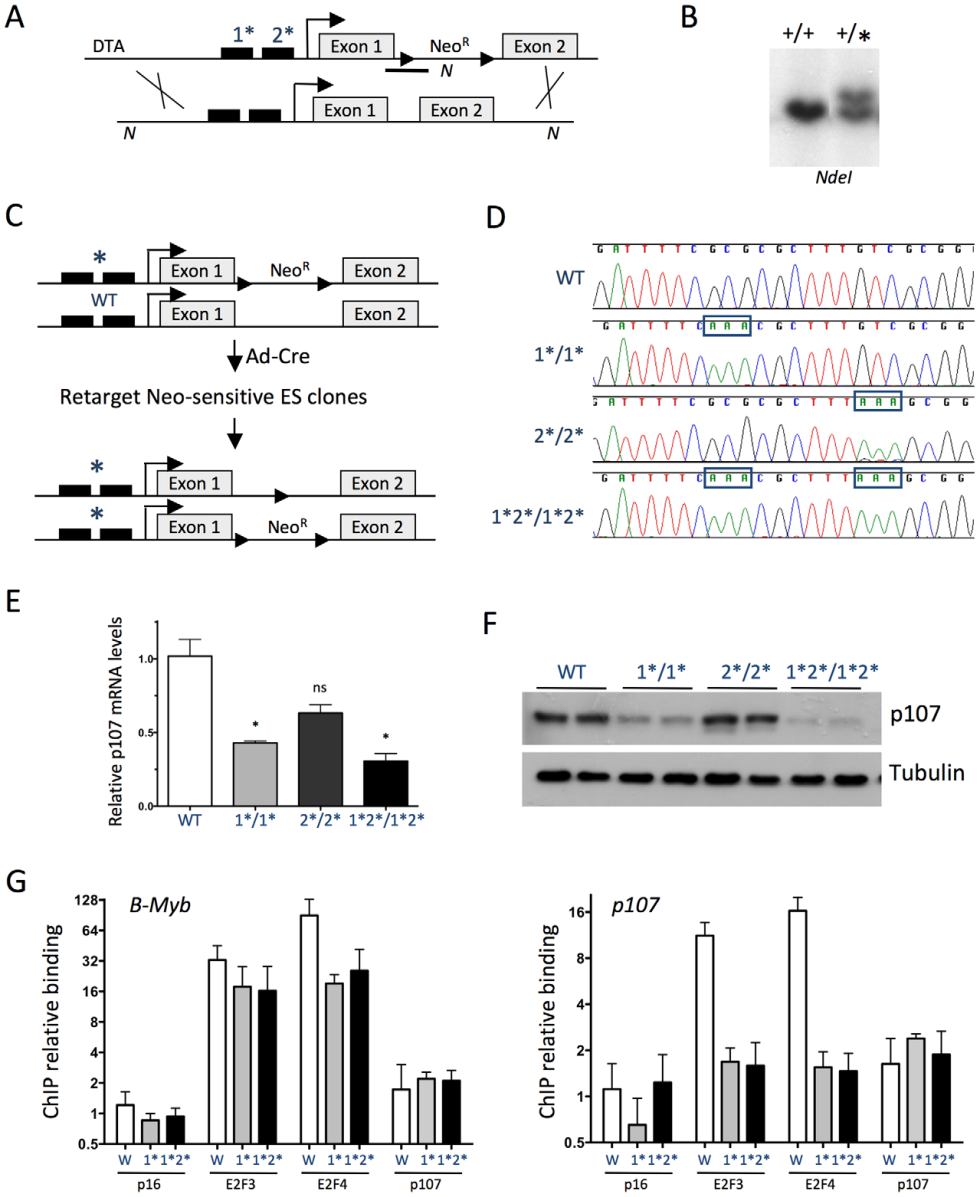
The E2F consensus binding sites in the *p107* promoter are bound by E2F family members and control *p107* expression in mESCs. (A) Schematic representation of the targeting construct (top) used to knock-in mutations into the E2F sites in the endogenous *p107* allele (bottom). Mutations in the E2F sites are indicated by asterisks. Neo^R^, neomycin resistance cassette; DTA, diphtheria toxin A. The black boxes indicate E2F consensus sites, the grey boxes indicate *p107* exons. (B) Representative Southern analysis for a wild-type mESC clone (+/+) and a correctly targeted allele (+/*). Genomic DNA was digested by *NdeI* (N in Figure 1A) and a 5′ internal probe spanning the junction between the *p107* intron and the Neo^R^ cassette was used (black line in Figure 1A). (C) Schematic representation of the strategy used to generate homozygous mutant mESCs. (D) Sequencing analysis of wild-type and homozygous mutant mESCs. The knock-in mutant sequences are marked by boxes. (E) RT-qPCR analysis of *p107* expression in wild-type and homozygous mutant cycling mESCs. *p107* mRNA levels were calculated relative to TATA-binding protein (*TBP*). (n = 4) (F) Immunoblot analysis of p107 expression in mESCs. Tubulin expression is shown as a loading control. (G) Quantitative chromatin immunoprecipitation (ChIP) analysis of E2F3 (n = 5), E2F4 (n = 5), and p107 (n = 2) on the *p107* promoter in wild-type (W) and homozygous mutant cycling mESCs. p16 antibodies serve as a negative control (n = 5). Fold enrichment was calculated over an unrelated DNA sequence (*actin*). The *B-Myb* promoter is shown as a control. The y-axis is plotted on a *log2* scale.

To examine the role of the consensus E2F binding sites in the regulation of the endogenous *p107* promoter, we generated a series of targeting vectors designed to knock-in the same series of mutations as those described for the luciferase vectors into the endogenous *p107* locus (Figure 2A). These vectors were electroporated into mESCs in order to generate heterozygous cells. Targeting was confirmed by both 5′ and 3′ Southern analysis, as well as by sequencing of amplified genomic DNA (Figure 2B and data not shown). Wild-type control mESCs were generated through targeting events in which the Neomycin resistance cassette was correctly targeted but the E2F binding sites remained untargeted. Targeted cells were then infected with an adenovirus expressing the Cre recombinase in order to remove the resistance cassette, followed by a second round of targeting (Figure 2C). This procedure generated homozygous cells of three knock-in genotypes: p107^E2F-1*/1*^ cells with mutations in the distal site of both *p107* alleles, p107^E2F-2*/2*^ cells with mutations in the proximal site, and p107^E2F-1*2*/1*2*^ cells with mutations in both E2F sites on both alleles of *p107*. Targeting was verified by Southern and sequencing analysis (Figure 2D and data not shown).

To determine the role of the consensus E2F binding sites in controlling endogenous *p107* expression, we first examined *p107* mRNA levels in control and homozygous mutant mESCs. In a pattern similar to the luciferase assays, quantitative RT-PCR (RT-qPCR) analysis showed that p107^E2F-1*/1*^ cells expressed 40-50% of the *p107* mRNA expressed by wild-type cells while p107^E2F-2*/2*^ cells expressed 70%; p107^E2F-1*2*/1*2*^ cells expressed the lowest amounts of *p107* in mESCs (Figure 2E). The relative levels of *p107* mRNA in these mutant cells reflected actual p107 protein expression (Figure 2F), providing additional evidence that p107 levels in these cells are regulated largely at the transcriptional level and suggesting that E2F activity is involved in this transcriptional control. Importantly, the basal *p107* promoter remained active despite the knock-in mutations, validating this knock-in approach to investigate the functional importance of discrete elements in the *p107* promoter.

We next examined if the point mutations introduced into the *p107* promoter affected the binding of E2F transcription factors and p107, the member of the Rb family with the highest level of expression in cycling cells [37], to the *p107* regulatory regions, an experiment that was not possible without the knock-in mutant cells. Using quantitative ChIP analysis, we found that both E2F3 and E2F4 bound to the wild-type *p107* promoter in mESCs (Figure 2G). The p107^E2F-1*/1*^ and p107^E2F-1*2*/1*2*^ cells demonstrated no binding for these two E2F family members to the *p107* promoter (Figure 2G), consistent with the decreased levels of *p107* expression observed in these cells (Figure 2E–2F). In all cases, there was significant binding of both E2Fs to the promoter of a control gene, *B-Myb* (Figure 2G). Rb family members are largely inactivated by hyperphosphorylation in undifferentiated mESCs [36]. As expected, we did not detect any significant binding of p107 on the *p107* or *B-Myb* promoters in wild-type or knock-in mutant cells under these conditions (Figure 2G) while p107 binding could be observed in asynchronously cycling mouse fibroblasts at the same promoter regions (data not shown). These experiments show that E2F transcription factors require the consensus E2F binding sites for binding to the *p107* promoter region. They also suggest that the distal E2F consensus binding site is the major mediator of E2F activity controlling *p107* transcription in cycling mESCs.

### *p107* expression is repressed in quiescent MEFs by Rb/E2F complexes via the tandem consensus E2F binding sites in the proximal promoter region

**Figure 3:**
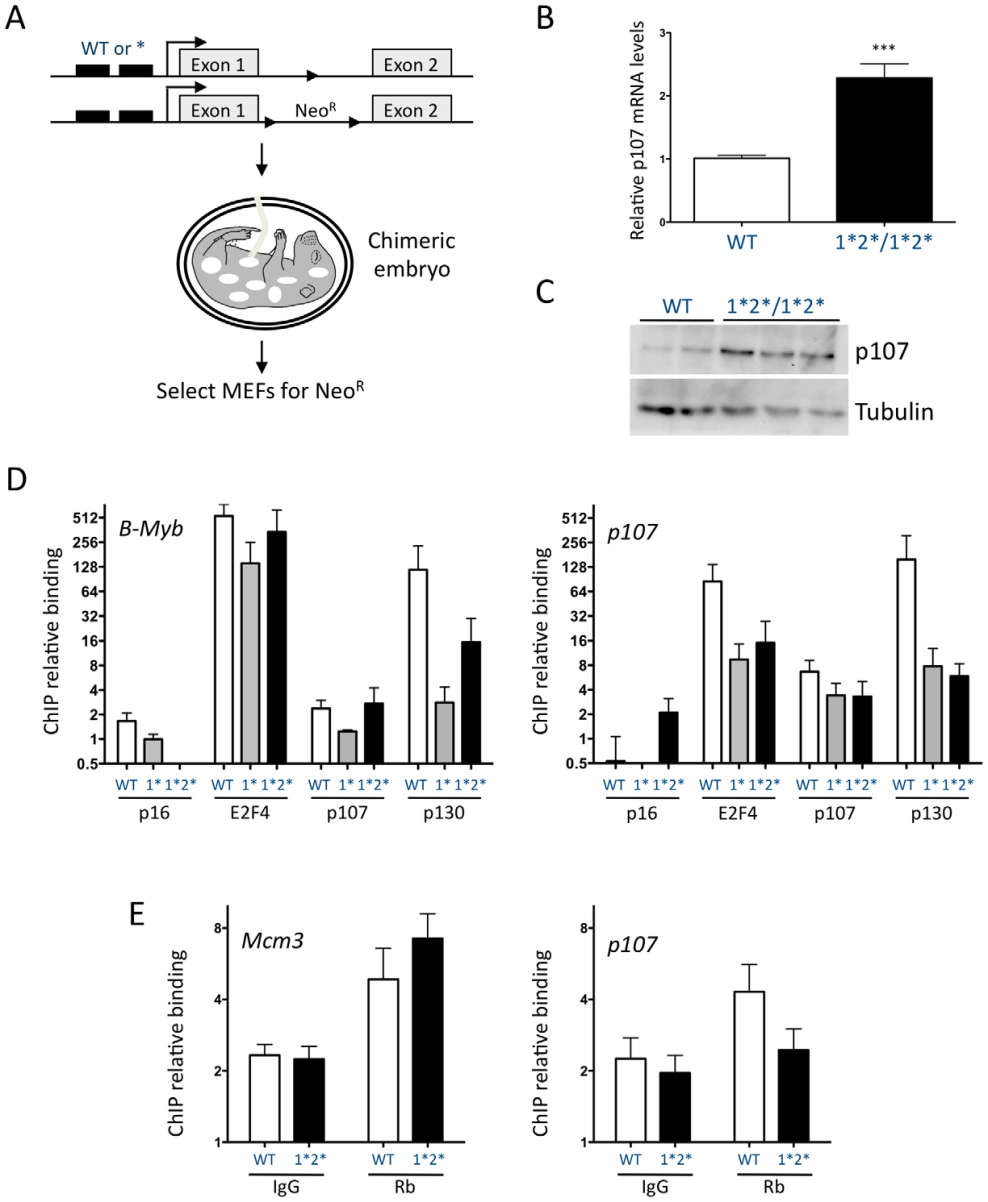
*p107* repression in quiescent MEFs is mediated by the two E2F binding sites. (A) mESCs targeted by the neomycin resistance cassette but retaining a wild-type *p107* promoter and mESCs targeted by homozygous mutations into the distal (1*/1*) or both E2F sites (1*2*/1*2*) were injected to generate chimeric embryos. Wild-type, p107^E2F-1*/1*^ and p107^E2F-1*2*/1*2*^ MEFs derived from chimeric embryos were selected for Neomycin resistance to generate pure populations. (B) RT-qPCR analysis of *p107* expression in quiescent wild-type and p107^E2F-1*2*/1*2*^ MEFs. (n≥9) (C) Immunoblot analysis of p107 in the same conditions. Tubulin expression is shown as a loading control. (D) Quantitative ChIP analysis of E2F4, p107, and p130 binding on the *p107* promoter in quiescent immortalized wild-type, p107^E2F-1*/1*^, and p107^E2F-1*2*/1*2*^ MEFs. The *B-Myb* promoter is shown as a control. (n = 3) (E) Quantitative ChIP analysis of Rb binding to the *p107* and *Mcm3* promoters in cycling immortalized wild-type and p107^E2F-1*2*/1*2*^ MEFs. Mouse IgG antibodies serve as a negative control. (n≥3) For (D,E), fold enrichment is calculated over *actin* and the y-axis is plotted on a *log2* scale.

To understand the function of the E2F binding sites in the control of *p107* during a more normal cell cycle and in G1 arrest, we grew MEFs from chimeric embryos generated from homozygous p107^E2F-1*/1*^, p107^E2F-1*2*/1*2*^, and control mESCs as indicated in Figure 3A. Due to lower numbers of chimeric embryos derived from the p107^E2F-1*/1*^ cells, experiments were only performed in this genotype after immortalization. Primary control and p107^E2F-1*2*/1*2*^ MEFs were first rendered quiescent in low serum. We found that p107^E2F-1*2*/1*2*^ MEFs expressed two times more *p107* mRNA than did the wild-type cells in the same conditions (Figure 3B). This fold increase, while somewhat variable depending on the MEF line (see below), was always observed and is similar to the increased levels of reporter activity observed with the p107-1*2* luciferase construct in quiescent MEFs (Figure 1D). A similar increase was observed with p107 protein levels in these cells (Figure 3C). These data showed that *p107* expression was repressed in G0 through the E2F binding sites present in its proximal promoter region. Accordingly, we found a significant decrease in E2F4 binding to the *p107* promoter in quiescent immortalized p107^E2F-1*/1*^ and p107^E2F-1*2*/1*2*^ MEFs compared to controls (Figure 3D), underscoring the role of the distal E2F binding site in the control of *p107* repression in G0-arrested cells. p107 levels are low in quiescent cells and we did not observe any significant binding of p107 to the *B-Myb* promoter in wild-type or p107^E2F-1*/1*^ and p107^E2F-1*2*/1*2*^ quiescent immortalized MEFs (Figure 3D). We found some binding of p107 to its own promoter in wild-type cells just above the non-specific signal found with the control antibody, and there was a trend towards decreased binding in the knock-in mutant cells (Figure 3D), even though p107 levels are higher in the mutant cells (Figure 3C). p130 binding to the *p107* promoter was decreased in p107^E2F-1*/1*^ and p107^E2F-1*2*/1*2*^ MEFs compared to controls; interestingly, p130 binding to the *B-Myb* promoter was also decreased in the mutant cells (Figure 3D). It is possible that increased p107 levels in the mutant quiescent cells may alter the composition of the protein complexes between members of the Rb and E2F families. One obvious candidate whose binding to the *p107* and *B-Myb* promoters could also be affected and could influence p130 binding in the mutant cells is Rb itself. Detection of murine Rb at the promoters of E2F target genes has proven challenging, especially in cells with low levels of Rb such as MEFs [29], [38]–[40]. While we have not measured Rb binding to the *p107* promoter in quiescent cells, we were able to detect Rb binding to the *p107* and *Mcm3* promoters in cycling immortalized MEFs. We found that Rb binding to the *p107* promoter was decreased to close to background levels in knock-in mutant cells but not changed at the promoter of the *Mcm3* gene (Figure 3E). Because of the low intensity and the variability of the ChIP signal for Rb, we cannot exclude that Rb may be binding to other sites in the *p107* promoter. However, altogether, these observations support a model in which Rb/E2F complexes bind to the *p107* promoter through the E2F consensus binding sites.

**Figure 4:**
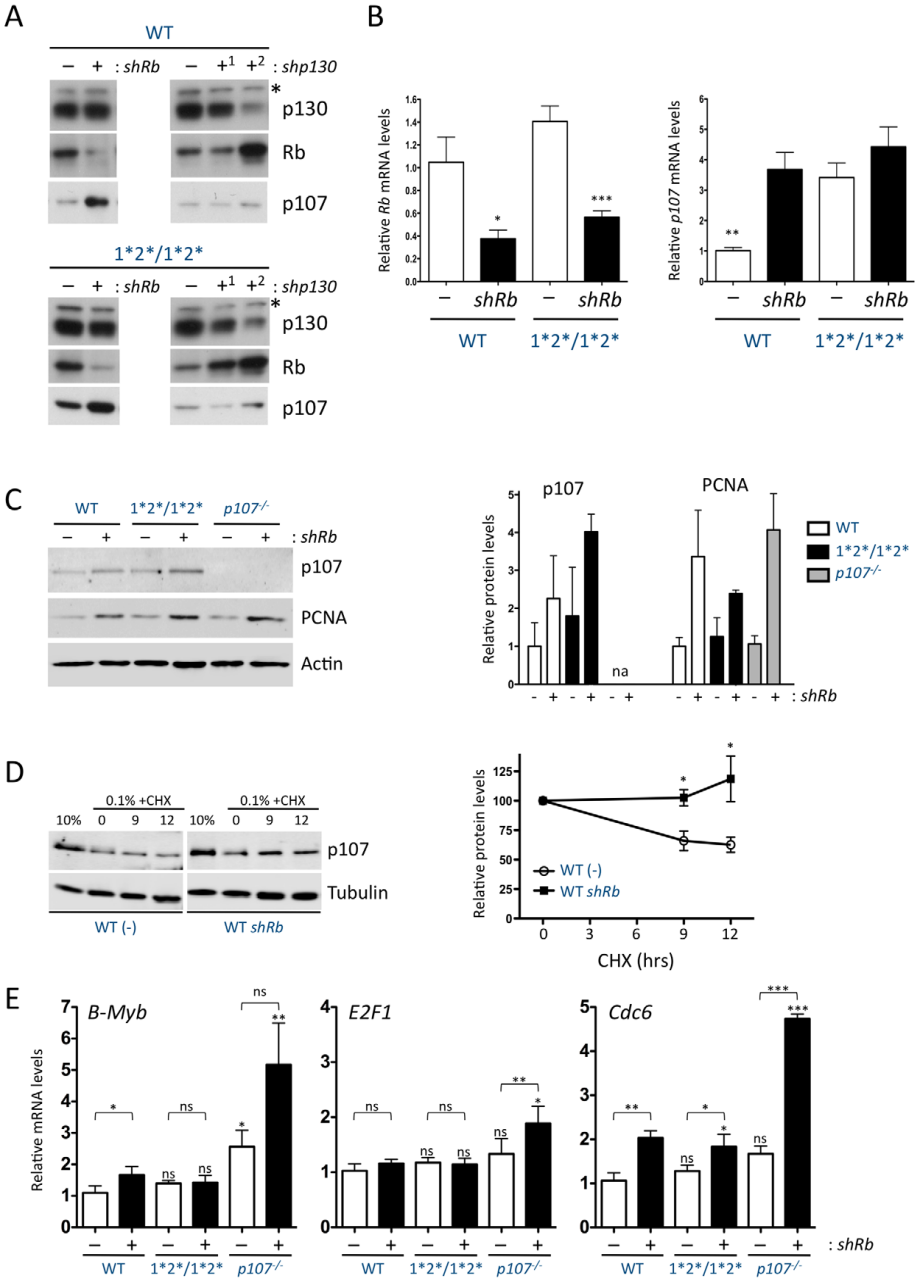
p107 levels increase in quiescent MEFs in the absence of Rb through transcriptional and post-translational mechanisms. (A) Top: Representative immunoblot analysis of p107, Rb, and p130 expression after knockdown of *Rb* (*shRb*) or *p130* (*shp130-1* and *shp130-2*) as compared to empty vector in primary quiescent MEFs. Bottom: same experiment in p107^E2F-1*2*/1*2*^ mutant MEFs. The asterisk shows a non-specific band that serves as a loading control; loading was also verified by Ponceau staining (not shown). (B) RT-qPCR analysis of *Rb* (left panel) and *p107* (right panel) mRNA relative to *TBP* in quiescent wild-type and p107^E2F-1*2*/1*2*^ MEFs infected with empty vector (−) or a vector to knock-down *Rb* (*shRb*). (n≥3) (C) Immunoblot analysis (left panel) of p107 levels in cells of the indicated genotypes infected with empty vector (−) or a vector to knock-down *Rb* (+). The E2F target PCNA serves as a positive control and β-Actin as a loading control. Protein quantification (right panel) is shown relative to β-Actin levels. p107 levels were not measured in *p107* mutant cells (na). (n = 2) (D) Immunoblot analysis (left panel) of p107 levels in quiescent wild-type MEFs infected with empty vector (−) or a vector to knock-down *Rb* (*shRb*) and treated with cycloheximide (CHX) for 9 and 12 hours (hrs). Quantification (right panel) is shown relative to Tubulin levels. (n = 2) (E) RT-qPCR analysis of *B-Myb* (left panel), *E2F1* (center panel), and *Cdc6* (right panel) mRNA levels relative to *TBP* in quiescent primary wild-type, p107^E2F-1*2*/1*2*^, and *p107^−/−^* MEFs after knockdown of *Rb* as in B. For statistical analysis, each MEF genotype was compared to the wild-type one by an unpaired Student's t-test, and the effect of *Rb* knockdown on each genotype was analyzed by a paired Student's t-test. (n≥3)

To determine whether the de-repression observed in the p107^E2F-1*2*/1*2*^ MEFs was due to the loss of a repression complex involving Rb family members, control and p107^E2F-1*2*/1*2*^ MEFs were infected with retroviruses stably expressing shRNA molecules directed against *Rb* or *p130* (Figure 4A, top). As previously shown [18], [20], *Rb* knock-down in quiescent MEFs resulted in an increase in p107 protein levels (Figure 4A, top). In contrast, we did not observe an increase in p107 expression in cells with a *p130* knock-down (Figure 4A, top, and data not shown). We could not functionally test if low levels of p107 expression altered its own transcription, although knockdown of *p107* has no effect on the expression of an eGFP transgenic reporter for *p107* in either cycling or quiescent MEFs ([20] and unpublished observations). Based on these observations, we sought to determine the consequences of knocking-down *Rb* in wild-type and p107^E2F-1*2*/1*2*^ cells for p107 levels. Expression of shRNA molecules in wild-type and mutant MEFs resulted in a significant knock-down of *Rb* mRNA levels (Figure 4B, left). As expected, decreased *Rb* levels led to increased *p107* mRNA levels in wild-type quiescent MEFs. In contrast, low levels of *Rb* did not result in a further de-repression of *p107* mRNA expression in p107^E2F-1*2*/1*2*^ MEFs (Figure 4B, right). Moreover, the degree of de-repression that occurred in wild-type cells upon Rb knockdown was similar to that seen through point mutations in the E2F binding sites (Figure 4B, right). These experiments strongly suggested that Rb represses *p107* through the two E2F binding sites in the *p107* promoter in quiescent MEFs.

In cycling mESCs (Figure 2E–2F) and in quiescent MEFs (Figure 3B and 3C), *p107* transcript levels correlate with p107 protein levels. In contrast, although quiescent wild-type MEFs with *Rb* knock-down and p107^E2F-1*2*/1*2*^ MEFs either with or without *Rb* knock-down all have similar *p107* mRNA levels (Figure 4B), our initial immunoblot analysis suggested that p107 protein levels were higher in p107^E2F-1*2*/1*2*^ MEFs with *Rb* knock-down compared to control wild-type cells with knock-down or knock-in mutant cells with wild-type *Rb* levels (Figure 4A). Additional experiments confirmed and quantified these observations (Figure 4C). In order to explore the potential post-transcriptional regulation of p107 levels in the absence of Rb, we treated wild-type MEFs in the presence and absence of *Rb* knockdown with cycloheximide, an inhibitor of translation. We found that p107 levels in the *Rb* knockdown cells remained more constant in the presence of cycloheximide than in wild-type cells treated with cycloheximide (Figure 4D). These data suggest that loss of Rb function may control p107 levels post-transcriptionally, at least in certain contexts. Nevertheless, these observations also support a model in which the transcriptional control of *p107* expression by Rb is largely through the two E2F binding sites in the *p107* promoter.

The increased levels of p107 protein found in quiescent p107^E2F-1*2*/1*2*^ MEFs that are further increased in the presence of *Rb* knockdown led us to ask what functional effect these increased levels of p107 may have on the transcription of other E2F target genes. We performed RT-qPCR analysis on several E2F target genes, and found that, as expected, the expression of some of the genes examined–*B-Myb*, *Cyclin A*, and *Cyclin E*–was increased in wild-type MEFs in which *Rb* has been knocked down (Figure 4E, left, shows the data for *B-Myb*, similar data for *Cyclin A* and *Cyclin E* are not shown); the expression of these same genes was not increased in p107^E2F-1*2*/1*2*^ MEFs in which *Rb* has been knocked down (Figure 4E, left). Interestingly, *Cdc6* expression was elevated in both wild-type and p107^E2F-1*2*/1*2*^ MEFs upon knock-down of *Rb*, although a much larger increase in *Cdc6* mRNA expression is observed in *p107^−/−^* MEFs with additional knockdown of *Rb* (Figure 4E, right). Lastly, *E2F1* expression was unchanged in wild-type and p107^E2F-1*2*/1*2*^ MEFs with or without *Rb* expression, but was de-repressed in *p107^−/−^* MEFs with *Rb* knockdown (Figure 4E, center). These results indicate that, in the absence of Rb, increased levels of p107 are able to repress the expression of some, but not all E2F target genes in quiescent MEFs.

### Activation of *p107* mRNA expression during cell cycle progression in MEFs is mediated by the tandem E2F binding sites

**Figure 5:**
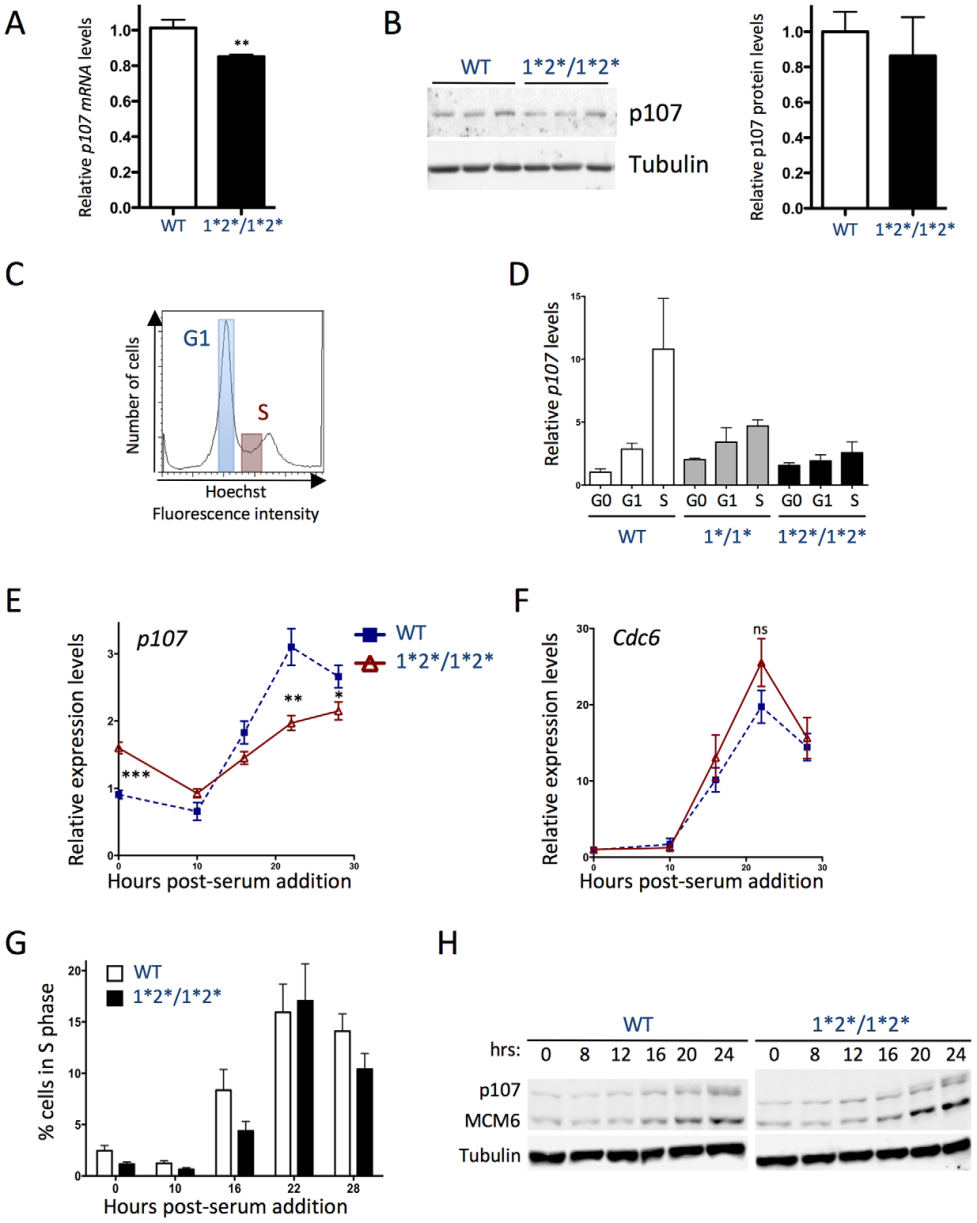
E2F binding sites mediate activation of the *p107* promoter in cycling cells. (A) RT-qPCR analysis of *p107* mRNA relative to *TBP* in asynchronously cycling primary wild-type and p107^E2F-1*2*/1*2*^ MEFs. (n = 12) (B) Immunoblot analysis (left panel) of p107 expression in wild-type and p107^E2F-1*2*/1*2*^ MEFs as in A. Tubulin expression is shown as a loading control. p107 protein quantification (right panel) is shown relative to Tubulin levels. (n = 3) (C) Representative example of Hoescht33342 staining of asynchronously cycling MEFs showing G1 and S phase populations; wild-type and mutant cells have similar profiles (data not shown). (D) RT-qPCR analysis of immortalized WT, p107^E2F-1*/1*^ and p107^E2F-1*2*/1*2*^ MEFs. For each genotype, G0 samples were collected after at least three days of serum starvation. Asynchronous cells were stained with Hoechst33342 and sorted by their DNA content into G1 and S-phase samples. (n≥2) (E) and (F) RT-qPCR analysis of primary wild-type and p107^E2F-1*2*/1*2*^ MEFs that have been synchronized in G0 by serum starvation. DMEM supplemented with 20% serum was added at time 0, and extracts were collected at 10 hrs, 16 hrs, 22 hrs, and 28 hrs post-stimulation. (E) *p107* mRNA and (F) *Cdc6* mRNA. n≥8 for both genotypes at all time points. (G) Percentage of cells in S-phase in primary MEFs collected during cell-cycle re-entry as in E. and F. Percentages were calculated by BrdU/PI analysis (n = 3). (H) Immunoblot analysis of p107 protein expression in primary wild-type and p107^E2F-1*2*/1*2*^ MEF extracts collected at 0 hr, 8 hrs, 12 hrs, 16 hrs, 20 hrs, and 24 hrs post-stimulation with 20% serum. MCM6 expression is shown as a positive control for cell cycle re-entry, and Tubulin levels are shown as a loading control. Note that the second, slowly migrating form of p107 at later time points probably reflects p107 phosphorylation during S phase.

We next investigated the role of the E2F binding sites in the cell-cycle dependent activation of *p107* transcription. We first found that asynchronously cycling p107^E2F-1*2*/1*2*^ MEFs expressed ∼10% less *p107* mRNA than did the wild-type cells (Figure 5A), a decrease which was barely observable at the protein level (Figure 5B). We speculated that because p107^E2F-1*2*/1*2*^ MEFs display a 1.5-2-fold de-repression of *p107* when they are in G0/G1, this could mask a decrease in *p107* expression at other phases of the cell cycle. To investigate this possibility, we expanded and stained control and mutant immortalized MEFs with Hoechst33342, a DNA intercalating agent that enabled the cells to be FACS-sorted by DNA content (Figure 5C). We found that wild-type G1 cells expressed 2–3 times more *p107* than did cells in G0, and this level increased up to 10-fold in S phase. On the other hand, while both p107^E2F-1*/1*^ and the p107^E2F-1*2*/1*2*^ cells showed some cell-cycle dependent induction of *p107*, this induction was lower than the induction of *p107* in wild-type cells during S-phase (Figure 5D).

To examine the regulation of *p107* transcription *via* the two E2F binding sites during cell cycle re-entry from G0, we synchronized primary wild-type and p107^E2F-1*2*/1*2*^ MEFs through serum starvation, and then stimulated cell-cycle re-entry through the addition of serum in the medium. We found that p107^E2F-1*2*/1*2*^ MEFs expressed higher levels of *p107* than wild-type cells initially and that the mutant cells failed to increase *p107* expression as much as the wild-type cells during cell cycle re-entry (Figure 5E), supporting the fact that the two E2F binding sites in the *p107* promoter are critical for *p107* up-regulation during S phase progression. Despite these differences in *p107* levels, MEFs of both genotypes re-entered the cell cycle with similar kinetics, as determined by measuring the mRNA levels of the highly cell cycle regulated gene *Cdc6* (Figure 5F) and by the two-dimensional analysis of DNA content by PI staining and BrdU incorporation (Figure 5G). One potential reason for the similarity of the cell cycle profiles in control and mutant cells is that p107 protein levels reached wild-type levels in p107^E2F-1*2*/1*2*^ mutant MEFs progressing through S phase (Figure 5H) in these re-entry experiments. These observations corroborated our findings in cells with *Rb* knock-down that other mechanisms exist to increase p107 levels in some contexts, beyond the control of *p107* transcriptional control by Rb/E2F complexes. Nevertheless, these experiments also confirmed that the transcriptional control of *p107* expression in cells re-entering the cell cycle is under the control of the two E2F binding sites in its proximal promoter region.

### Lower levels of *p107* specifically during S phase are sufficient to accelerate the cellular proliferation

**Figure 6:**
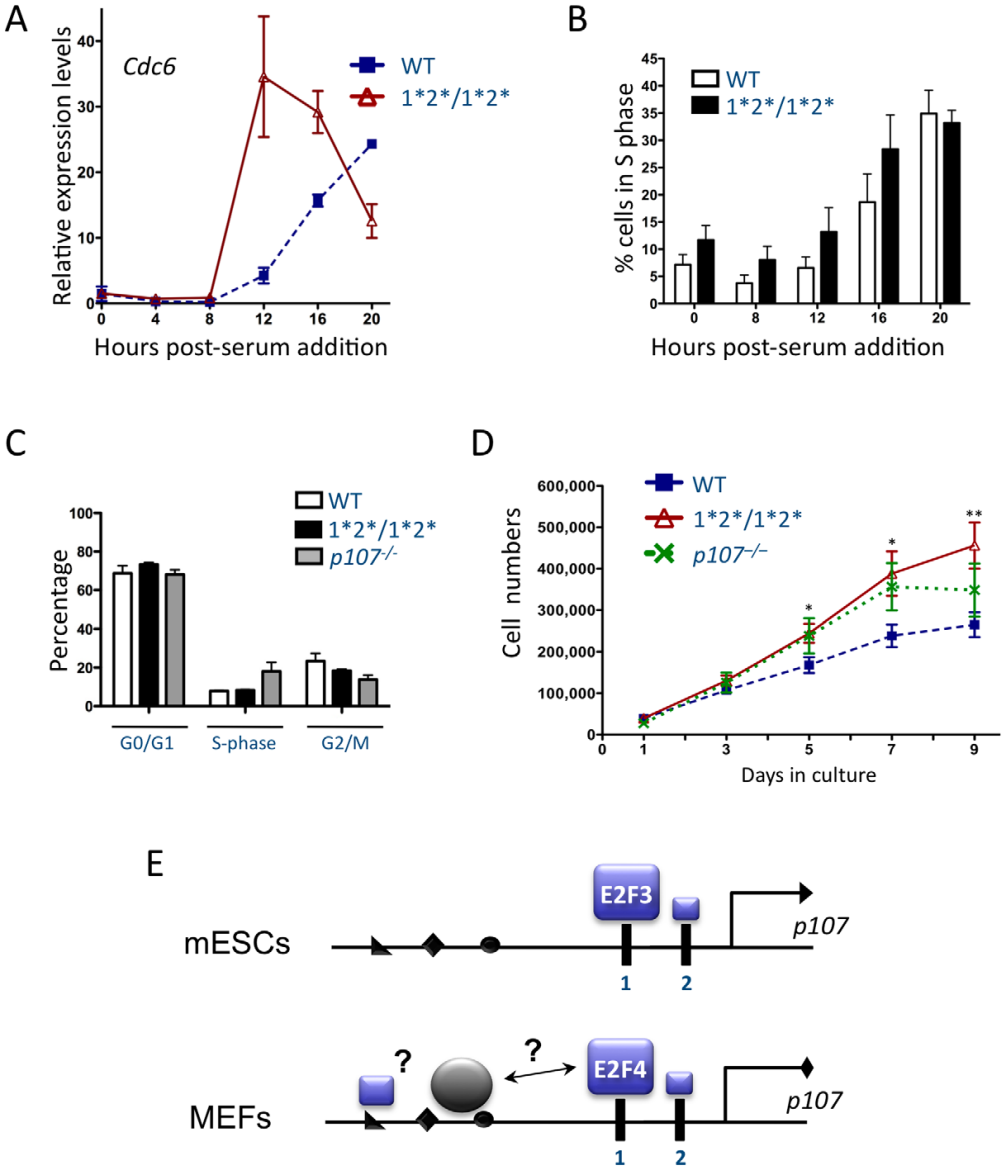
Altered *p107* expression affects cellular proliferation. (A,B) Immortalized wild-type and p107^E2F-1*2*/1*2*^ MEFs were synchronized in G0 through at least three days of serum starvation. DMEM supplemented with 20% BGS was used to stimulate cell-cycle entry. Extracts were collected at the number of hours indicated post-serum stimulation. (A) RT-qPCR analysis of *Cdc6* mRNA in wild-type and p107^E2F-1*2*/1*2*^ MEFs. (n = 3) (B) Percentage of cells in S-phase, as determined by BrdU/PI staining, at the indicated time points. (n≥4) (C) Cell-cycle profiles of asynchronous primary wild-type, p107^E2F-1*2*/1*2*^, and *p107^−/−^* MEFs. Percentages of cells in each phase were determined by BrdU/PI staining. (n≥2) (D) Cellular proliferation of primary wild-type, p107^E2F-1*2*/1*2*^, and *p107^−/−^* MEFs. Equal numbers of cells were plated at day 0. Cells were then counted every other day from day 1 to day 9 post-plating. For statistical analysis, p107^E2F-1*2*/1*2*^ cells were compared to wild-type cells at each time point. (n≥13) (E) Model for the context-dependent regulation of *p107* transcription by E2F family members. In cycling mESCs, activating members of the E2F family such as E2F3 bind to the *p107* promoter mostly through the distal consensus E2F binding site (site 1). In quiescent MEFs, binding of the E2F4 repressor is also largely dependent on the presence of the distal consensus site. However, E2F4 may also be recruited to the *p107* promoter through interactions with other transcription factors and/or by binding to other DNA sequences. The size of the E2F boxes indicates the relative binding activity.

The lower levels of *p107* RNA found in p107^E2F-1*2*/1*2*^ cells at the G1/S transition and during DNA replication provided a system to test the role of *p107* specifically during S phase progression. As discussed above, we found that the kinetics of *Cdc6* induction were largely similar between primary wild-type and p107^E2F-1*2*/1*2*^ mutant MEFs, with only a slight increase in *Cdc6* maximal levels and a slight acceleration of *Cdc6* induction in the mutant cells (Figure 5F). Interestingly, however, when we repeated the same experiment with MEFs immortalized through knockdown of *p19^ARF^*, we found that p107^E2F-1*2*/1*2*^ mutant MEFs induced *Cdc6* mRNA levels more rapidly (Figure 6A), suggesting that these mutant MEFs re-entered the cell cycle more quickly. BrdU/PI analysis further suggested that immortalized p107^E2F-1*2*/1*2*^ mutant MEFs generally entered S phase earlier than control MEFs in this context (Figure 6B). Together, these results suggest that *p107* plays a critical role in controlling the kinetics of entry into S-phase in immortalized cells.

These defects in cell cycle re-entry led us to investigate if altered *p107* levels may change the length of the cell cycle in asynchronously proliferating primary cells. To investigate the importance of the regulation of *p107* transcription for cell cycle control, we performed cell proliferation assays comparing wild-type, p107^E2F-1*2*/1*2*^, and *p107^−/−^* MEFs. BrdU/PI analysis of these asynchronously growing MEFs did not reveal any significant differences in the cell cycle profiles between the wild-type and p107^E2F-1*2*/1*2*^ MEFs, although slightly more knock-in mutant cells were in G0/G1 and slightly fewer in G2/M than wild-type cells within this analysis; significantly more *p107^−/−^* MEFs were in S-phase than in either of the other genotypes (Figure 6C). Despite this absence of difference in BrdU incorporation, we found that p107^E2F-1*2*/1*2*^ MEFs proliferated similarly to *p107^−/−^* MEFs and more rapidly than wild-type cells (Figure 6D). Thus, decreased levels of *p107* mRNA specifically during S phase and in asynchronously cycling cells, even in primary MEFs, are sufficient to recapitulate the phenotype of *p107^−/−^* MEFs. Together, these observations indicate that p107 plays an important role at specific points during S phase in mammalian cells.

## Discussion

The classical view of the E2F family of transcription factors is that they are necessary to drive cell-cycle progression by binding to the promoters of and activating genes necessary for S phase, including those needed for nucleotide synthesis and DNA replication (reviewed in [3], [41]). However, it has also long been observed that the list of E2F target genes also includes negative regulators of the cell cycle, including *p16*, *Rb*, and *p107*. Traditional knock-out studies obscure the careful balance of these positive and negative feedback loops and the relative importance of each individual target. The knock-in of potential binding sites for specific transcription factors has not been extensively used [42], [43] but proves here to be an extremely informative approach to dissect the functional role of specific nodes in complex regulatory networks. Our experiments demonstrate that E2F family members and Rb control *p107* transcription largely through two tandem E2F binding sites in the proximal promoter of the *p107* gene. Our data also identify functional differences between the two sites and the E2F activity bound to these sites in different contexts (see model in Figure 6E).

E2F transcription factors make up a diverse family whose members can all recognize the same consensus sequence. While some E2F transcription factors activate target gene expression, others repress transcription, either dependent upon or independent from their association with Rb family members. Many E2F target genes, like *p107*, reveal an even more complex promoter structure that includes two, or sometimes more, E2F consensus sites, and each site could have distinct functions in the control of the target gene [44]–[46]. Why certain promoters have several E2F binding sites and what dictates if a site serves to repress or activate transcription is not understood. In the specific example of *p107*, our data show that the distal E2F binding site is favored by both E2F3 and E2F4 in mESCs, indicating that activating and repressor E2Fs may act through the same binding site *in vivo*. Our data also indicate that binding to the consensus site is context-dependent: while E2F4 binding in mESCs requires the presence of at least one of the two binding sites, an E2F4 binding activity is still retained in MEFs with mutations in both E2F binding sites. This observation suggests the existence of secondary E2F binding sites and/or the presence of co-factors, including Rb family members, which may help tether E2Fs to a promoter region (Figure 6E). This residual binding activity may explain why *p107* levels are still increasing during cell cycle re-entry in p107^E2F-1*2*/1*2*^ mutant MEFs. In the future, the availability of cells with knock-in mutations in individual binding sites in the *p107* promoter will provide novel tools to dissect how transcription factors and chromatin-remodeling enzymes interact with Rb/E2F complexes to regulate the expression of E2F target genes in different cellular contexts.

Similar to the residual binding of the E2F transcription factors to the mutant *p107* promoter, we observed above background levels of Rb, p130, and p107 bound to the *p107* promoter in p107^E2F-1*2*/1*2*^ mutant MEFs. Rb family members have been well described to bind to many other protein binding partners, including ATF-1 [31] and Sp1 [30]. The mouse *p107* promoter contains consensus sequences for both ATF and Sp1 (Figure 1B), and we cannot exclude that these sites, or others, may mediate E2F-independent binding of all three Rb-family members to the *p107* promoter. Interestingly, we also found that the p107^E2F-1*2*/1*2*^ mutant MEFs demonstrated reduced binding of p130 to the *B-Myb* promoter during quiescence. Expression analysis of *B-Myb* did not reveal substantial de-repression in these mutant MEFs, and increased levels of p107 were able to repress *B-Myb* expression in the absence of Rb. Together these results are consistent with the model that all three Rb family members are able to regulate *B-Myb*, and that these complexes may shift in response to altered Rb family levels. Furthermore, the trend towards increased Rb binding to the *Mcm3* promoter in the p107^E2F-1*2*/1*2*^ mutant MEFs (Figure 3E) was observed at other promoters (*B-Myb* and *Cyclin A*, data not shown), which may further suggest that p130 is displaced by Rb in these cells.

The tandem E2F consensus sites in the human *p107* promoter had previously been demonstrated to have differential functions over control of *p107* [27]. In contrast, our experiments show that the distal site is most important for both activation and repression of the *p107* promoter in mouse cells. This discrepancy could be explained by the presence of a single point mutation in the mouse promoter, which makes the proximal site a less perfect E2F consensus site. We found that correcting the proximal site in the mouse *p107* promoter (TTTG**T**CGC → TTTG**G**CGC) did increase the activity of this promoter in luciferase assays approximately 3 fold relatively to the parental construct (unpublished data). However, in the absence of an intact distal site, the activity of even the “corrected” construct was substantially lower than that of a wild-type construct, further emphasizing the relative importance of this site in the mouse *p107* promoter. These results also suggest that the human promoter, with two perfect E2F consensus sites, should generally be more responsive to E2F transcription factors than the mouse promoter. Therefore, this discrepancy alone is unlikely to explain why p107 is up-regulated and able to compensate for loss of *Rb* function in the mouse retina but not the human retina [47] or why mice and patients with an *Rb* mutation develop a distinct tumor spectrum. While the E2F sites are highly conserved across mammalian species, the rest of the *p107* promoter is not, and these other evolutionary changes may further impact promoter regulation by E2F and other factors. Interestingly, in humans, single nucleotide polymorphisms (SNP) of unknown frequency have been identified in the *p107* promoter, including one that deletes one of the 4 Ts in the distal E2F binding site; while this may not affect E2F binding (only 3 Ts are required in the consensus sequence), this polymorphism may potentially alter the physical orientation of the two sites relative to each other and influence *p107* transcription. Future experiments will continue to dissect the mechanisms regulating *p107* transcription, including the interactions between E2F family members, Rb family members, and other transcription factors that bind to the *p107* promoter. These interactions may also help to explain the tissue-specificity of expression of *p107 in vivo* [20], [48].

While several binding partners for p107 and its expression profile in various cell types are well known, the unique cellular functions of p107 are still poorly understood [2], [49]. Overexpression of p107 can arrest some cell types in G1 [50], but loss of *p107* function often results in no visible phenotypes, probably because of functional compensation by Rb and p130 [13], [17]. Interestingly, loss of *p107* in neural progenitors results in the activation of Notch signaling and increased proliferation [51], [52]. *p107^−/−^* mice on a BALB/cJ background also display a myeloid hyperplasia and mutant MEFs derived from these mice demonstrate accelerated proliferation [53]. Furthermore, an insertional mutagenesis screen for tumor suppressor genes identified *p107* as a tumor suppressor in B-cell lymphoma [54]. However, the mechanisms underlying these loss-of-function phenotypes are still unclear. Some studies have suggested that p107 may have a particular function during the progression from late G1 to S phase [55], [56], when p107 levels are highest. We show here that expression of abnormally low levels of *p107* during S phase results in increased cell numbers in a proliferation assay. Importantly, while the p107 protein is still expressed and *p107* levels found during S phase in p107^E2F-1*2*/1*2*^ cells are similar to those found in wild-type cells in G0/G1, and while BrdU incorporation failed to detect a significant difference between knock-in mutant cells and wild-type cells, the consequences for the cell cycle are as strong as in *p107* null cells. This observation indicates that p107 levels are probably critical during very specific stages of the cell cycle, including during the DNA replication phase, and this role for p107 during S phase should be the focus of future studies to elucidate the cellular functions of this cell cycle regulator.

Unexpectedly, in immortalized MEFs in which *p19^ARF^* has been knocked-down, the importance of *p107* expression during S-phase is even more evident, as the kinetics of cell cycle re-entry are altered in the p107^E2F-1*2*/1*2*^ cells compared to wild-type cells, whereas these kinetics are unchanged in primary MEFs. This difference between the function of p107 during S phase in immortal and primary MEFs is potentially related to an additional function of p19^ARF^ during S phase [57]. This function could stem from a network of interactions between p107, p19^ARF^, E2F1 and c-Myc during S phase [58]–[63] and these findings could reveal cooperation between appropriate expression of *p107* and *p19^ARF^* in the control of S phase.

In the absence of Rb, de-repression and/or activation of *p107* transcription is thought to result in higher levels of p107 that may then suppress the functions of activating E2F family members [64], [65]. It is interesting to note that a similar mechanism has evolved independently in plants [66] and that this type of negative feedback loop also exists in the control of the cell cycle in yeast [67], strongly suggesting that this genetic circuitry is a universal component of the regulatory networks ensuring proper cell cycle progression. Here, we investigated the mechanisms by which this feedback loop is activated in mammalian cells using a mouse genetics approach.

While it has long been hypothesized that direct regulation of the *p107* promoter by Rb is the mechanism by which compensatory upregulation of p107 occurs in the absence of Rb, our mutant cells enabled us to distinguish effects of Rb loss on transcriptional and post-transcriptional compensatory expression of p107. Recently it has been shown that the p107 protein may be more stable in non-cycling human hepatocellular carcinoma cell lines when Rb is absent than when Rb is present [68]. Similarly, we found that in quiescent MEFs, the p107 protein becomes more stable when *Rb* is knocked-down, suggesting that transcriptional regulation alone may not fully explain compensatory levels of the p107 protein. Clearly, this increase in p107 protein levels in cycling cells or *Rb* mutant cells independent of *p107* transcription could serve as a feedback mechanism to limit the effects of decreased Rb levels. The relative contribution of transcriptional and post-transcriptional mechanisms of p107 up-regulation in different contexts will be the focus of future studies.

We hypothesized that disrupting the transcriptional feedback loop between Rb/E2F and p107 would prevent compensation in the absence of Rb, such that the low *p107* levels observed in p107^E2F-1*2*/1*2*^ MEFs would cooperate with decreased Rb expression, potentially recapitulating some of the phenotypes observed in *Rb*;*p107* double knock-out MEFs. Additional cell cycle assays comparing primary and immortalized knock-in mutant MEFs, both with and without Rb knock-down, failed to reveal conditions in which the knock-in mutation would cooperate with loss of Rb in allowing cells to grow in conditions that were permissive to growth of *Rb;p107* double knock-out MEFs (data not shown). These results suggest that, in most contexts, the lower levels of *p107* observed specifically during S-phase are insufficient to overcome the higher levels of *p107* observed at other stages of the cell cycle, at least in terms of reducing the ability of p107 to compensate in the absence of Rb.

Instead, we found that the increased levels of p107 observed in quiescent MEFs were able to repress expression of some genes in the absence of Rb even better than the increased levels of p107 found in wild-type MEFs in the absence of Rb. Interestingly, the ability of increased levels of p107 to compensate for absence of Rb in repressing E2F target genes during quiescence depends on the particular gene. Increased levels of p107 protein produced by transcriptional de-repression and increased protein stability resulted in repressed levels of *B-Myb*, *Cyclin A*, and *Cyclin E* but had no further effect on *Cdc6* (Figure 4E and data not shown). These results are consistent with several studies suggesting that the Rb family members regulate both distinct and overlapping target genes [23], [69], [70]. These observations also suggest that further increasing p107 levels in *Rb* mutant cells through a variety of mechanisms may enhance the compensatory abilities of this Rb family member.

A remaining question is why humans develop retinoblastoma upon loss of Rb while mice do not. It has recently been demonstrated that mouse retinal progenitors deficient for *Rb* display increased levels of *p107* mRNA whereas human retinal progenitors do not increase the amount of *p107* expressed when *Rb* is knocked down in culture [47]. This observation, and the fact that *Rb/p107* double mutant mice develop retinoblastoma [71] supports the idea that p107 levels are important for its tumor suppressor activity in *Rb*-deficient retinal cells. However, no system has been devised to test whether the increased expression of *p107* observed in Rb mutant cells directly contributes to the ability of p107 to compensate for the loss of Rb, or if this increased transcription is merely coincidental to a constitutive overlapping function shared by Rb and p107. In other words, it is possible that basal levels of *p107* in *Rb* mutant cells would be sufficient to suppress retinoblastoma development. A similar question can be asked in other cell types in which *p107* loss of function by knock-out enhances tumor development in *Rb* mutant cells and during development [13], [15], [72]. Our results indicate the converse, i.e. that even higher levels of p107 may more completely compensate in the absence of Rb, in terms of target gene expression. The generation of mESCs and MEFs in which the E2F binding sites in the *p107* promoter were singly mutated were intended to discretely separate the ability of *p107* to be activated in the absence of Rb from other sources of transcriptional regulation. However, these genetic studies clearly demonstrated that in MEFs, a single E2F site is critical for both the repression of *p107* in non-cycling cells and the activation of *p107* in cycling cells, making the separation of these two activities impossible. In future experiments, mice carrying point mutations in the E2F binding sites in the *p107* promoter or mice carrying fragments of the human *p107* promoter may help explore the necessity of p107 up-regulation in the prevention of retinoblastoma in mice by distinguishing between overlapping and compensatory expression patterns of *p107*.

## Materials and Methods

### Ethics statement

Mice were maintained according to practices prescribed by the NIH at Stanford's Research Animal Facility accredited by the AAALAC.

### Generation of knock-in *p107* BAC and targeting vectors

Recombineering in bacteria [73] was used to generate the p107^E2F-1*^, p107^E2F-2*^, and p107^E2F-1*2*^ BACs. Briefly, BAC clone RP23-163J20 ordered from BACPAC (http://bacpac.chori.org/) was transformed into the EL250 strain and the heat-inducible recombinase present in this strain enabled the insertion of a Neomycin resistance cassette into intron 1 of *p107* by homologous recombination. Approximately 500 bp of the wild-type promoter sequence was cloned into pBluescript. Mutation of the E2F sites was performed using blunt end primers carrying the wild-type sequence or the point mutations. The 500 bp fragments were then targeted to the *p107* BAC with the conditional Neomycin resistance cassette. To generate the targeting vectors, we excised approximately 4 kb upstream of the E2F binding sites and 4 kb downstream of the Neomycin resistance cassette from the BAC DNA into a targeting vector backbone carrying a DTA cassette.

### Cell culture

mESCs and MEFs were cultured as described previously [74]. For expression analysis in asynchronous MEFs, 3×10^5^ cells were plated per 6 cm culture dish. For BrdU/PI analysis in asynchronous MEFs, 2.5×10^5^ cells were plated per 6 well. Extracts were collected 48 hours later. For quiescent cell analysis were plated at higher density: 8×10^5^ per 6 cm dish, 3×10^5^ per 6 well, or 1.5×10^5^ per 12 well. The following day cells were washed twice with PBS and then cultured in DMEM supplemented with 0.1% serum for at least 72 hours. For extracts from synchronized MEFs, cells were rendered quiescent and cultures were re-stimulated with DMEM supplemented with 20% serum as above after at least 72 hours in low serum conditions. Extracts were collected at various time points after stimulation. For proliferation assays, MEFs were plated at either 2.5×10^4^ or 5×10^4^ cells per 12 well on day 0. Cells were counted in duplicate and were given fresh media every other day.

For cycloheximide experiments, wild-type MEFs were rendered quiescent for 3 days as above. At time 0, cycloheximide (Calbiochem, 25 mg/ml in methanol) or methanol was added to cells at a concentration of 30 µg of cycloheximide per ml of DMEM with 0.1% serum.

### Luciferase assays

To construct the plasmid reporters, primers were designed to amplify the *p107* promoter from the targeted BAC clones (wild-type and mutants) to enable cloning directly into the multiple cloning site of PGL3-basic (Promega). The reverse primer was positioned to include all sequence upstream of the translation start site. 1.4×10^4^ mESCs (V6.5) and 1.25×10^4^ MEFs were plated in wells of 48-well plates and transfected one day later. For mESCs, luciferase activity was read two days after transfection following the manufacturer's instructions (Promega). For quiescent MEFs, luciferase activity was read 24 hours after the withdrawal of serum. In all luciferase assays, 500 ng of each *p107* construct was co-transfected with 125 ng of a Renilla luciferase vector. For exogenous E2F3 experiments, 100 ng of DP1 (a gift from the Dyson lab) was co-transfected with either 100 ng of pCDNA empty vector or 100 ng of CMV-E2F3. Transfections were carried out using the Fugene6 Reagent (Roche).

### Lentiviral and retroviral infections

*Rb* knock-down was achieved using the pSicoR lentivirus [75], as described [18]. The sequence in the *Rb* cDNA that is targeted by the shRNA molecules is 5′-TGAGAGCAAGGATGTCTCA-3′. *p19^ARF^* and *p130* knock-down was achieved using the pSiripp retrovirus [18], as above. The sequence in the mouse *p19* cDNA that is targeted by the shRNA molecules is: 5′-CACCGGAATCCTGGACCAG-3′. The sequences in the mouse *p130* cDNA that is targeted by the shRNA molecules are: 5′-TCACTCTGCTCTGTTACGT-3′ and 5′- GATGTGGCGAATGACCGAG-3′.

### Generation of mESCs and MEFs with mutations in the *p107* promoter

DTA- p107^E2F-1*^ and DTA- p107^E2F-1*^ ^2*^ targeting vectors were electroporated into V26.2 mESCs (C57BL/6) and DTA- p107^E2F-2*^ was electroporated into J1 mESCs (129Sv/J). Genomic DNA from targeted clones was screened by 5′ and 3′ Southern analysis, details of which are available upon request. The E2F binding sites of clones targeted with the neomycin resistance cassette were sequenced using the primers described for *p107* promoter ChIP below. Clones that were appropriately targeted by the neomycin resistance cassette but that retained wild-type binding sites were used as wild-type controls. Heterozygous mESCs for each construct were infected with Ad-Cre and plated for single colonies. Colonies were picked and screened for neomycin sensitivity. E2F binding sites were again sequenced to rule out loss a larger loss of the chromosome. Clones retaining heterozygous sequence for the binding site(s), as well as the loxP site in intron 1 were re-targeted and rescreened by Southern analysis and sequencing.

p107^E2F-1*/1*^ and p107^E2F-1*2*/1*2*^ homozygous mutant mESCs and controls (described above) were injected into wild-type blastocysts by the Stanford Transgenic Research Facility. MEFs were derived 11 days post-implantation. Pure populations were obtained through selection with 600 µg/ml of Geneticin (Invitrogen). MEFs were generated from one p107^E2F-1*/1*^ clone, two independently targeted p107^E2F-1*2*/1*2*^ clones, as well as two independent control clones. Where indicated, immortalized MEFs were generated through retroviral infection with a vector that expresses shRNA molecules against *p19^ARF^* [18].

### RNA and protein analysis

RNA was extracted from frozen cell pellets with TRIzol (Invitrogen). TaqMan or SYBR green quantitative PCR was performed as described previously [20], [76]. Rb, p107, TBP, and CDC6 primers were described previously [76], [77]. Other primer sequences are as follows: for *B-Myb*, forward primer, 5′-TTA AAT GGA CCC ACG AGG AG-3′ and reverse primer, 5′-TTC CAG TCT TGC TGT CCA AA-3′; for *E2F1*, forward primer, 5′-TGC CAA GAA GTC CAA GAA TCA-3′ and reverse primer, 5′-CTT CAA GCC GCT TAC CAA TC-3′. All relative expression analyses were calculated relative to *TBP* (TATA binding protein).

Immunoblots were detected as described previously [74]. Quantitative immunoblot analysis was performed using an Odyssey Infrared Imager from LI-COR Biosciences. Antibodies used were as follows: rabbit anti-p107 (sc-318, Santa Cruz Biotechnology), mouse anti-Rb [78], mouse anti-p130 (BD 610621), goat anti-MCM6 (sc-9843), and mouse anti-PCNA (sc-56). Loading was verified with antibodies against mouse anti-alpha-Tubulin (Sigma T9026), by anti-β-Actin (Sigma A5441), or by staining of total protein with Ponceau.

### Chromatin immunoprecipitation

Quantitative chromatin immunoprecipitation (ChIP) for E2F3, E2F4, p107, and p130 was performed as described previously [76]. Rb ChIP was performed as described in the Farnham laboratory protocol (http://genomics.ucdavis.edu/farnham/pdf/FarnhamLabChIP%20Protocol.pdf) with a few modifications. Chromatin was sonicated using a Virsonic probe sonicator at setting 2 at 20% output power for 8 cycles of 15 seconds. The chromatin was pre-cleared before being diluted and bound by 4 µg of the primary antibody overnight at 4°C. Each ChIP was then incubated with 8 µg rabbit anti-mouse IgG (MP Biomedicals #55436) as a secondary for 1 hour. The nucleoprotein complexes were pulled down by Pansorbin cells (Calbiochem, Cat# 507862). The DNA was digested with Proteinase K and RNaseA and then purified by a Qiagen QIAquick PCR Purification Kit. Additional details are available upon request.

Antibodies used for immunoprecipitations were as follows: E2F3 (sc-878X), E2F4 (sc-1082X), p107 (sc-318X), p130 (sc-317X), p16 (sc-467), Rb [78], and normal mouse IgG (sc-2025).

*p107*, *B-Myb*, and *Mcm3* promoter binding were assessed through quantitative PCR using SybrGreenER Mastermix (Invitrogen). *p107* forward, 5′-GGT CCA TCT TCT TAT CCC ATT CCG-3′; *p107* reverse, 5′-CTT CGG GGT TTT CTT TTC CCT C-3′; *B-Myb* forward, 5′-CTC GTG TCT TGT ACG CTT CGC C-3′; *B-Myb* reverse, 5′-CAC GTT CCC AGG AAC TGC AGC T-3′; *Mcm3* forward, 5′- AGC CAA TCA TAA CGC GTC TC-3′; *Mcm3* reverse, 5′-CAG CTC CAC ATC ATC CAG CA-3′; *actin* forward: 5′-GCT TCT TTG CAG CTC CTT CGT TG-3′; *actin* reverse, 5′-TTT GCA CAT GCC GGA GCC GTT GT-3′.

### Cell cycle assays

For primary and immortal MEF synchronized cell cycle analysis, cells were pulsed with BrdU for 2 hours prior to trypsinization. For primary MEF asynchronous cell cycle analysis, cells were pulsed with BrdU for 4 hours. BrdU and propidium iodide staining and analysis was performed as described previously [79], and analyzed on a BD FACSCalibur instrument. Data was analyzed using FlowJo software (Tree Star).

Subconfluent, immortalized MEFs were trypsinized and resuspended in DMEM +10% serum at 10×10^6^ cells/ml. Hoechst33342 was added at 30 mg/ml and incubated at 37°C for 1 hour in the dark. Cells were spun and resuspended in 1 ml of DMEM with fresh Hoechst, strained through a 40 mm cell strainer and then sorted at the Stanford Shared FACS Facility.

### Statistical analysis

Statistical significance was assayed by unpaired Student's t-test, except where otherwise indicated. *: p-value<0.05; **: p-value<0.005; ***: p-value<0.0005; ns: not significant. Mean and standard error are shown.
